# Clinicopathological and Prognostic Value of Gastric Carcinoma Highly Expressed Transcript 1 in Cancer: A Meta-Analysis

**DOI:** 10.1155/2020/6341093

**Published:** 2020-08-26

**Authors:** Xi Zhou, Yanghua Fan, Yu He, Anna Mou, Fu Wang, Yong Liu, Zhen Wu

**Affiliations:** ^1^Department of Orthopedics, Peking Union Medical College Hospital, Chinese Academy of Medical Sciences, Peking Union Medical College, Beijing, China; ^2^Department of Neurosurgery, Beijing Tiantan Hospital, Capital Medical University, Beijing, China; ^3^Department of Plastic Surgery, Plastic Surgery Hospital, Chinese Academy of Medical Sciences, Peking Union Medical College, Beijing, China; ^4^Department of Radiology, Sichuan Academy of Medical Sciences, Sichuan Provincial People's Hospital, Chengdu, Sichuan Province, China; ^5^Department of Orthopedic Surgery, Shandong Provincial Hospital Affiliated to Shandong University, Ji'nan, Shandong, China

## Abstract

**Background:**

Long noncoding RNA gastric cancer highly expressed transcript 1 (lncRNA GHET1) is often reported to be abnormally expressed in multiple cancers, but the situation is different in different cancers. Therefore, a meta-analysis is necessary to clarify the value of lncRNA GHET1 as a prognostic indicator in cancer.

**Methods:**

Relevant research studies on lncRNA GHET1 and cancer were retrieved from three electronic literature databases of Web of Science, PubMed, and OVID. Meanwhile, hazard ratios (HRs) and 95% confidence intervals (CIs) were calculated to explore the relationship between lncRNA GHET1 expression and survival of cancer patients. The odds ratios (ORs) and 95% CIs were calculated to assess the association of lncRNA GHET1 expression with pathological parameters of cancer patients.

**Results:**

The meta-analysis included a total of 11 studies involving 714 cancer patients. The pooled HR suggests that high lncRNA GHET1 expression is associated with poor overall survival. In addition, high expression of lncRNA GHET1 was found to be associated with larger tumor size, poor histological grade, high tumor stage, lymph node metastasis, and distant metastasis.

**Conclusions:**

High lncRNA GHET1 expression can predict poor survival and pathological parameters. And lncRNA GHET1 could serve as a new indicator in multiple cancers.

## 1. Background

Cancer is still the main disease that threatens people's lives and health. The American Cancer Society estimates new cancer cases and deaths in the USA every year, and 1,806,590 new cancer cases and 606,520 cancer deaths are expected in the USA according to Cancer Statistics in 2020 [1]. Nevertheless, cancer is a genetically heterogeneous disease with a poor prognosis. Finding new gene therapy targets is still a hot topic.

Long noncoding RNA (lncRNA) is a type of RNA with a length >200 nt and lacking or no open reading frame (ORF). The number of lncRNA is much lower than that of encoding protein genes (mRNA). More and more studies show that lncRNA has important functions such as transcription and epigenetic regulation in diseases [2, 3]. At the same time, recent studies have shown that lncRNA is closely related to the occurrence, development, invasion, metastasis, and prognosis of cancer [4–7], and lncRNA may be used as a new cancer treatment marker [8–10].

lncRNA gastric carcinoma highly expressed transcript 1 (lncRNA GHET1, AK123072), a recently identified lncRNA discovered in 2014, is located on chromosome 7q36.1 in the human genome [11]. In the meantime, GHET1 is found to play a critical role in the progression of gastric carcinoma, which is achieved through promoting the c-Myc stability. An increasing number of studies have been carried out to examine the role of lncRNA GHET1 in cancers, which reveal that lncRNA GHET1 is dysregulated in multiple cancers and that it plays an important role in tumor growth, metastasis, and invasion, as well as poor patient survival [11, 12]. lncRNA GHET1 may affect tumor metastasis and prognosis; however, a majority of existing studies are limited by their small sample sizes and discrete outcomes. As a consequence, an updated meta-analysis was performed in this study to determine the prognostic value of lncRNA GHET1 in cancer patients.

## 2. Materials and Methods

### 2.1. Literature Collection

First, this systematic review and meta-analysis is reported in accordance with the Preferred Reporting Items for Systematic Review and Meta-Analysis (PRISMA) Statement. Then, articles regarding lncRNA GHET1 as a prognostic biomarker for the survival of cancer patients were systematically retrieved in three online databases (PubMed, Web of Science, and OVID) from inception to January 20, 2019, by two authors independently in accordance with the standard guidelines for meta-analyses [13, 14]. Text word and MeSH strategy were adjusted according to the database in this retrieval, which included the following terms (“Long non-coding RNA Gastric Carcinoma High Expressed Transcript 1” or “Gastric Carcinoma Proliferation Enhancing Transcript 1” or “LncRNA GHET1”) and (“recurrence” or “outcome” or “survival,” “cancer” or “neoplasm” or “tumor” or “carcinoma,” “prognosis” or “prognostic”). Additionally, the reference lists of the relevant articles were also manually retrieved to prevent any missed articles.

### 2.2. Study Selection

All the included studies were evaluated, and data were extracted by two authors independently. The study inclusion criteria were as follows: (1) studies in which all tumors were confirmed by histological or pathological examinations; (2) studies in which lncRNA GHET1 expression in human tumor tissues was measured and patients were grouped in accordance with the lncRNA GHET1 expression level; (3) studies that performed statistical analyses on the pathological or patient survival parameters concerned with lncRNA GHET1 expression, such as overall survival (OS), high tumor stage (HTS), lymph node metastasis (LNM), or larger tumor size (LTS); and (4) studies with sufficient original data.

Besides, the study exclusion criteria were as follows: nonhuman studies and non-English studies; editorials, reviews, and expert opinions as well as letters; database analysis without original data; and studies concerning the functions of lncRNA GHET1 only but without the molecular mechanism analysis.

### 2.3. Data Extraction

Relevant data were examined and extracted from the original articles by two reviewers independently. Any disagreement between them was solved through the consensus with a third reviewer. A series of data were collected for this meta-analysis, including surname of the first author, publication year, country, tumor type, sample size, number of patients with LTS, poor histological grade (PHG), HTS, LNM, and distant metastasis (DM), reference gene and detection method of lncRNA GHET1, HRs and 95% CIs of elevated lncRNA GHET1 for OS, threshold of lncRNA GHET1 expression level, and the Newcastle–Ottawa scale (NOS) score.

### 2.4. Statistical Methods

The Stata version 12.0 software was adopted for all statistical analyses. In addition, the *Q* and *I*^2^ tests were conducted to detect the potential heterogeneity, the results of which indicated significant heterogeneity in this meta-analysis (*I*^2^ ≥ 50% and *P* < 0.1) [15]. Typically, a fixed- or a random-effects model should be adopted according to the results of heterogeneity analysis. In our meta-analysis, the random-effects model should be adopted in the presence of significant heterogeneity among the studies. Afterwards, the potential publication bias was also assessed by Egger's test and Begg's funnel plot. Notably, the aggregated ORs and HRs should also be extracted from the published data, and the crude HRs should be adopted if they could be obtained directly from the publications. For HRs and the corresponding 95% CIs that were not directly reported in the studies, the survival data extracted from Kaplan–Meier curves would be utilized to estimate the HRs. To summarize the survival outcome, both the SE and the log HR should be adopted [16]. Moreover, 95% CIs and ORs were pooled to assess the relationships of clinicopathological parameters (such as LTS, DM, LNM, and HTS) with lncRNA GHET1.

## 3. Results

### 3.1. Study Characteristics

**Figure 1:**
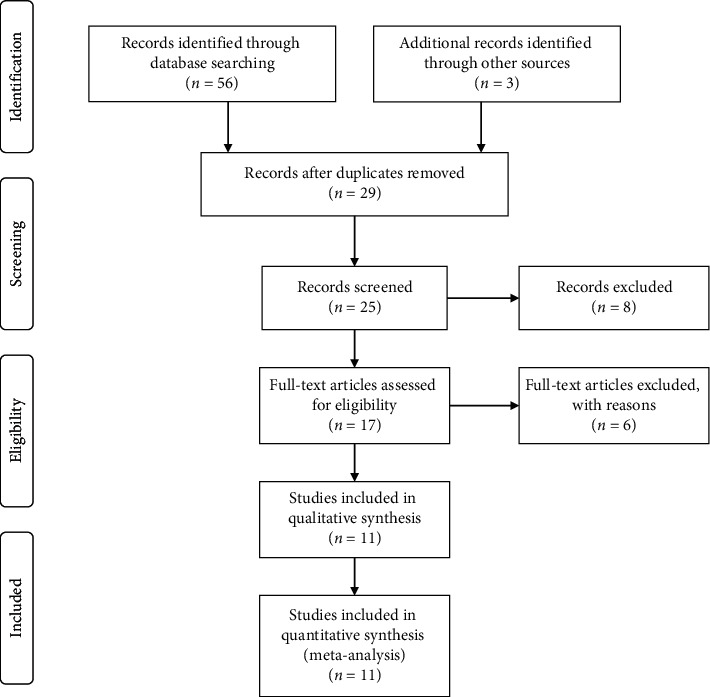
Flowchart presents the steps of study selection in this meta-analysis.

Details of the study selection process are displayed in Figure 1. Eleven studies involving seven hundred and fourteen patients were enrolled in this meta-analysis according to the study exclusion and inclusion criteria [11, 17–26].  Table 1 summarizes the features of the eleven studies included in this meta-analysis. Typically, the sample size in these studies ranged from 42 to 105, with an average of 64.9. Besides, all the enrolled studies were published between 2014 and 2018 and were carried out in China. Among these studies, respective one study had focused on hepatocellular carcinoma (HCC) [18], bladder cancer (BC) [19], esophageal squamous cell carcinoma (ESCC) [20], head and neck cancer (HNC) [21], breast cancer (BRC) [23], osteosarcoma (OSC) [25], and pancreatic cancer (PC) [26], while two focused on non-small-cell lung cancer (NSCLC) [17, 22], and two concentrated on gastric cancer (GC) [11, 24]. All clinicopathological parameters were dependent on pathology. Concretely, GAPDH was found to be the reference gene of lncRNA GHET1 in all these studies.

### 3.2. Association of lncRNA GHET1 Expression with Survival

**Figure 2:**
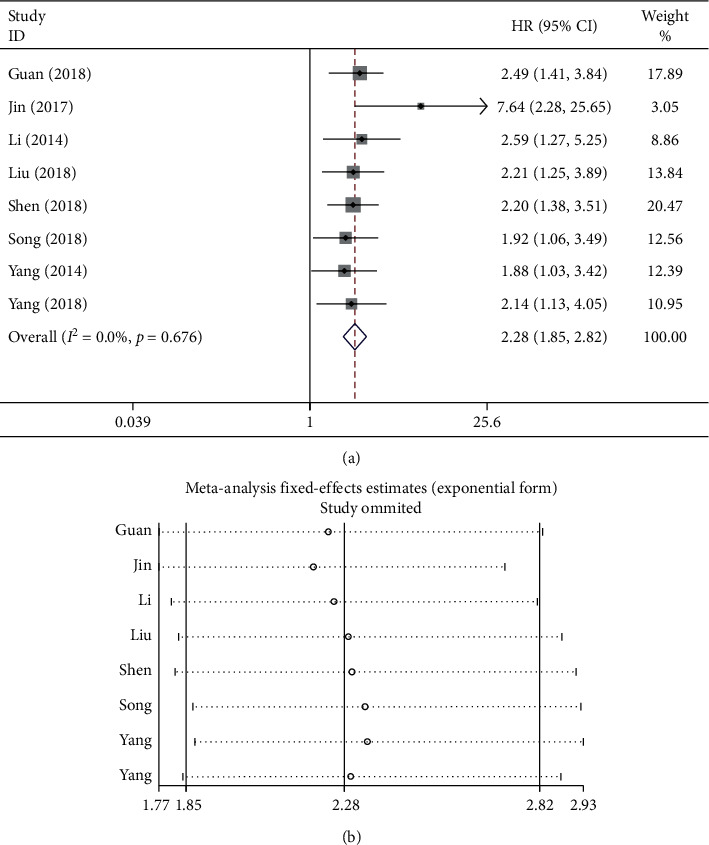
Forest plot (a) and sensitivity analysis (b) show the relationship between lncRNA GHET1 expression and OS in cancer.

**Figure 3:**
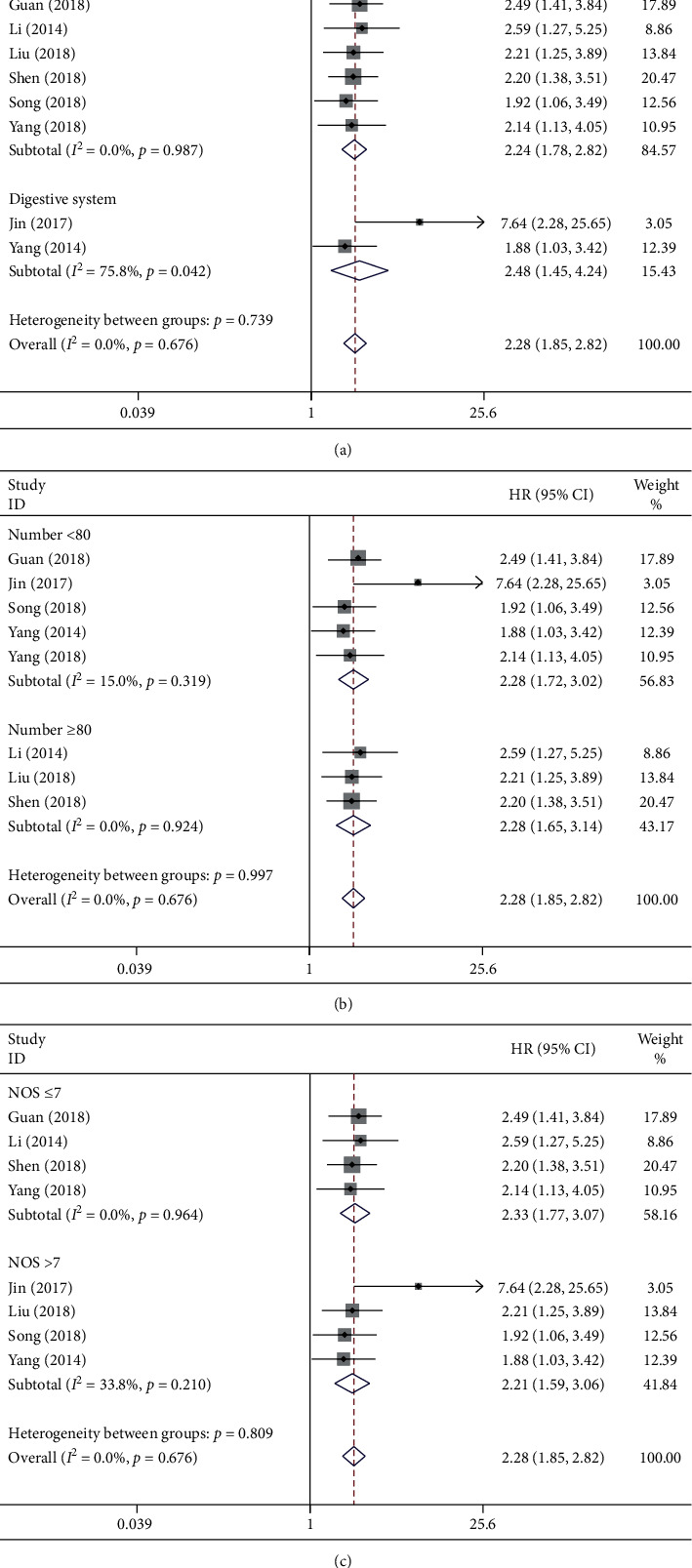
Forest plots of subgroup analysis for OS of cancer patients: subgroup analyses by tumor type (a), sample size (b), and NOS score (c).

To assess the function of lncRNA GHET1 in the OS for cancer patients, the cumulative meta-analysis was carried out in this meta-analysis. In addition, the relationship of OS with lncRNA GHET1 was reported in eight studies involving 553 patients (Table 2). Typically, the fixed-effects model was adopted since there was no significant heterogeneity (*I*^2^ = 0.0% and *P*_*Q*_ = 0.676). Our results indicated that OS was evidently related to lncRNA GHET1 (pooled HR = 2.28, 95% CI: 1.85–2.82; Figure 2(a)) in cancer patients. Besides, a sensitivity analysis was also performed, which had confirmed the robustness of these results (Figure 2(b)). Subsequently, subgroup analyses were further conducted based on the cancer type, sample size, and NOS score, and the results were consistent with those described previously (Table 3 and Figure 3).

Taken together, these results revealed that a shorter OS might be associated with high lncRNA GHET1 expression in cancer patients; as a result, lncRNA GHET1 might serve as an independent factor of survival for cancer patients.

### 3.3. Association of the lncRNA GHET1 Expression Level with LTS

**Figure 4:**
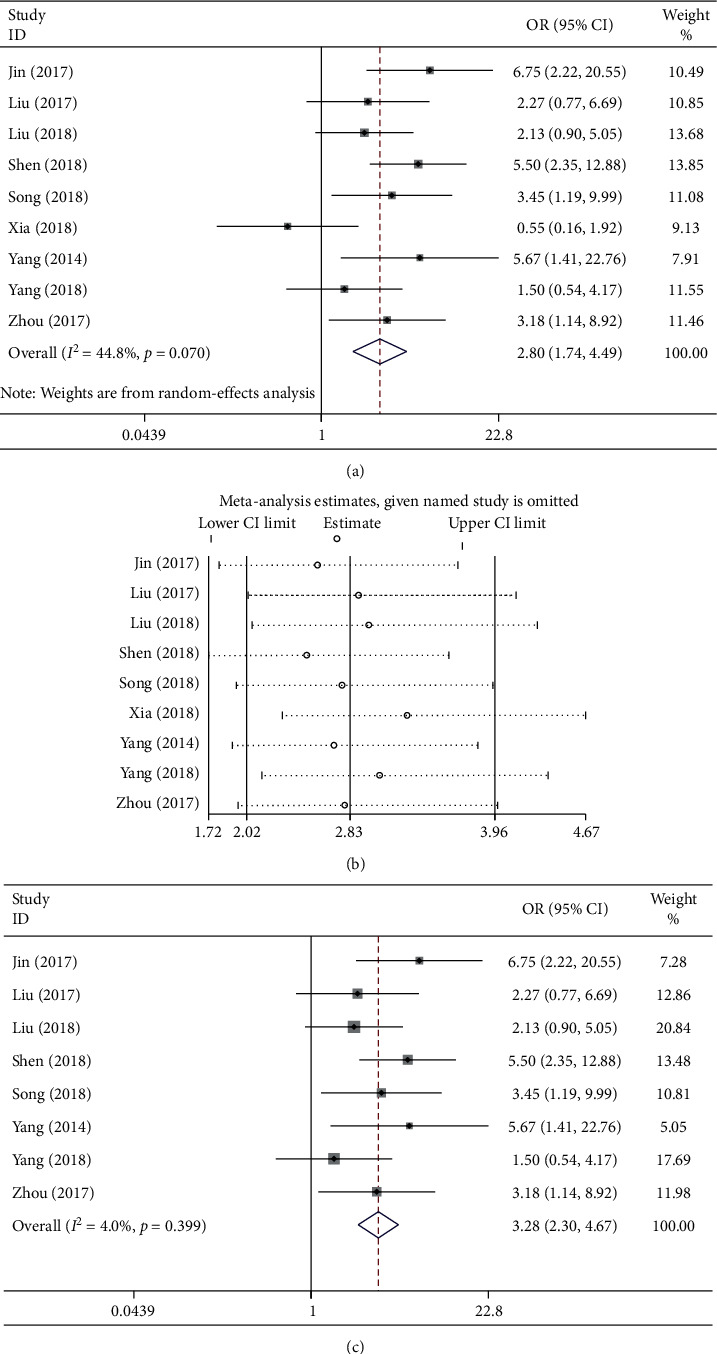
Forest plot (a) and sensitivity analysis (b, c) show the association between LTS and lncRNA GHET1 expression in cancer.

Figure 4(a) shows the association between LTS and lncRNA GHET1 expression identified from nine studies recruiting 582 patients. The random-effects model was adopted since there was a significant heterogeneity among these studies (*I*^2^ = 44.8% and *P*_*Q*_ = 0.070). The pooled OR was 2.80 upon analysis (95% CI: 1.74–4.49; high versus low lncRNA GHET1 expression). Afterwards, a sensitivity analysis was carried out among all the included studies, and the heterogeneity had disappeared after excluding the study by Xia et al. (*I*^2^ = 4% and *P*_*Q*_ = 0.399), with the OR between high versus low lncRNA GHET1 expression groups of 3.28 (95% CI: 2.30–4.67) (Figures 4(b) and 4(c)).

In accordance with these results, a significant difference was noted between the two groups. As far as the cancer patients were concerned, high lncRNA GHET1 expression could remarkably predict a higher risk of LTS.

### 3.4. Association between the lncRNA GHET1 Expression Level and PHG

**Figure 5:**
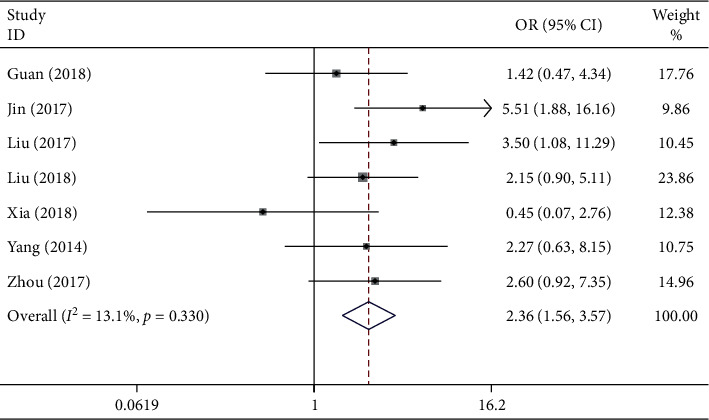
Forest plot reveals the relationship between lncRNA GHET1 expression and PHG in cancer.

In this meta-analysis, data regarding the association between the lncRNA GHET1 expression level and PHG were collected from the 409 cancer patients recruited in seven eligible studies. The fixed-effects model was adopted since no obvious heterogeneity was detected (*I*^2^ = 13.1% and *P*_*Q*_ = 0.330). The OR between the high and low lncRNA GHET1 expression groups was 2.36 (95% CI: 1.56–3.57; Figure 5). Consistently, a significant difference was detected in the incidence of PHG between these two groups, suggesting a significant relationship between the risk of PHG and high lncRNA GHET1 expression.

### 3.5. Association between the lncRNA GHET1 Expression Level and HTS

**Figure 6:**
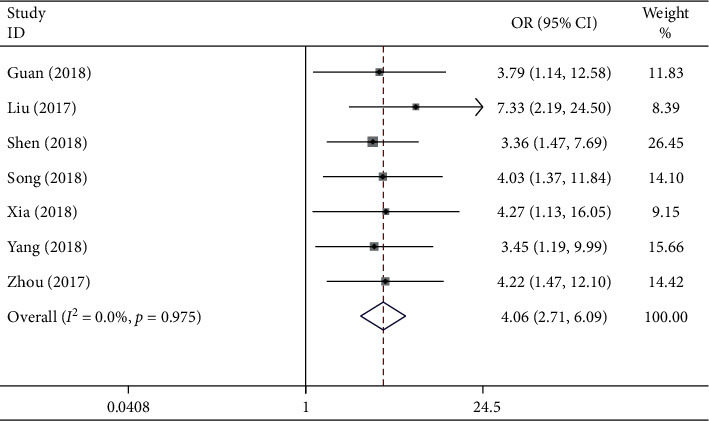
Forest plot presents the association between HTS and lncRNA GHET1 expression in cancer.

In this meta-analysis, the correlation of HTS with lncRNA GHET1 expression was explored in seven eligible studies involving 438 patients. The fixed-effects model was selected (*I*^2^ = 0.0% and *P*_*Q*_ = 0.975) in the absence of obvious heterogeneity. Our results indicated that HTS in cancer patients was associated with high lncRNA GHET1 expression (pooled OR = 4.06 and 95% CI: 2.71–6.09; Figure 6). Our results suggested that, compared with the low lncRNA GHET1 expression group, the HTS in the high expression group was evidently upregulated, demonstrating that the risk of HTS was notably correlated with high lncRNA GHET1 expression.

### 3.6. Association between the lncRNA GHET1 Expression Level and LNM

**Figure 7:**
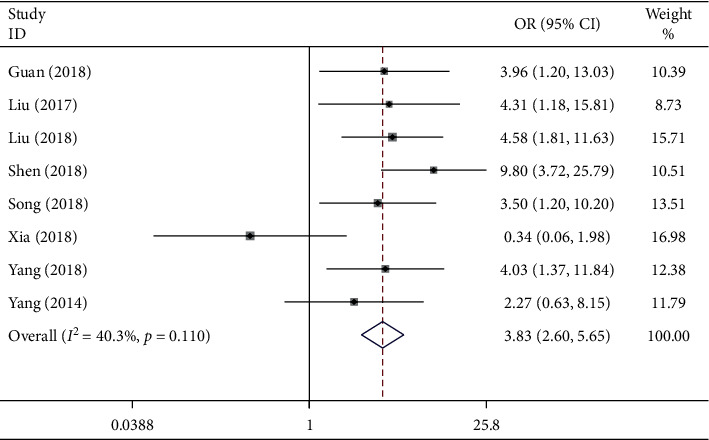
Forest plot displays the relationship between LNM and lncRNA GHET1 expression in cancer.

In this meta-analysis, data collected from the eight eligible studies involving 502 cancer patients would be examined. Similarly, the fixed-effects model would be adopted since there was no obvious heterogeneity (*I*^2^ = 40.3% and *P*_*Q*_ = 0.110). The OR of the high versus low lncRNA GHET1 expression groups was 3.83 (95% CI: 2.60–5.65; Figure 7). Accordingly, a significant difference in the incidence of LNM was also discovered between these two groups. For cancer patients, high lncRNA GHET1 expression could well predict the high risk of LNM.

### 3.7. Association between the lncRNA GHET1 Expression Level and DM

**Figure 8:**
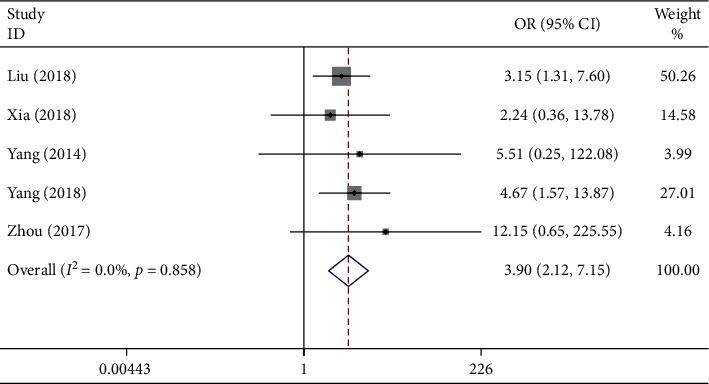
Evaluation of the relationship between lncRNA GHET1 expression and DM in cancer.

In this meta-analysis, the correlation of HTS with lncRNA GHET1 expression was examined in five eligible studies recruiting 294 patients. The fixed-effects model was selected in this study since there was limited heterogeneity (*I*^2^ = 0.0% and *P*_*Q*_ = 0.858). In addition, the OR of high versus low lncRNA GHET1 expression groups was 3.90 (95% CI: 2.12–7.15; Figure 8). Accordingly, a significant difference was detected in the incidence of DM between these two groups, which revealed that high lncRNA GHET1 expression could significantly predict a higher risk of incidence of DM in cancer patients.

### 3.8. Publication Bias

**Figure 9:**
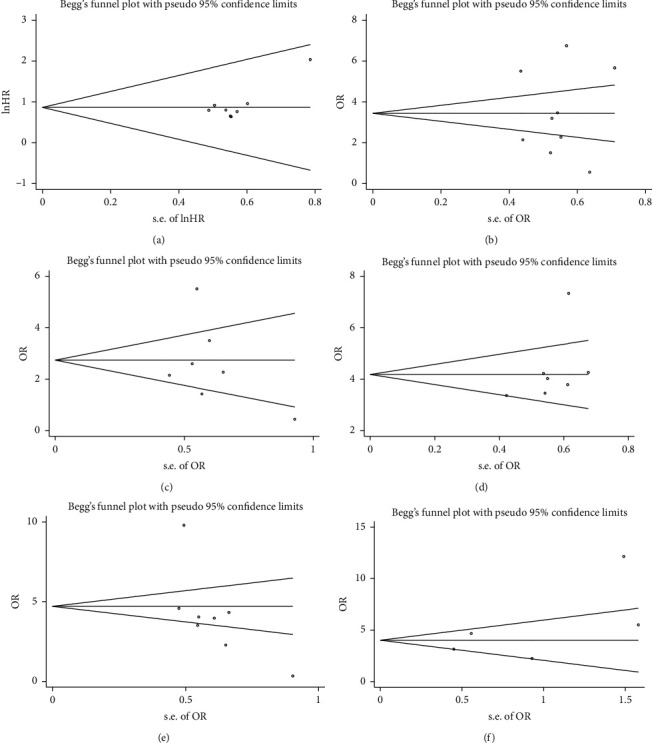
Begg's and Egger's publication bias plots of all eligible studies evaluating the relationship between lncRNA GHET1 expression and OS (a), LTS (b), PHG (c), HTS (d), LNM (e), and DM (f).

To evaluate the potential publication bias, Begg's funnel plot and Egger's test were carried out in this meta-analysis. As could be observed from Figure 9, there was no evidence of obvious asymmetry for OS (Pr > |*z*| = 0.536; Figure 9(a)), LTS (Pr > |*z*| = 0.917 and *P *> |*t*| = 0.984; Figure 9(b)), PHG (Pr > |*z*| = 0.548 and *P *> |*t*| = 0.608; Figure 9(c)), HTS (Pr > |*z*| = 0.133 and *P *> |*t*| = 0.243; Figure 9(d)), LNM (Pr > |*z*| = 0.174 and *P *> |*t*| = 0.096; Figure 9(e)), and DM (Pr > |*z*| = 0.462 and *P *> |*t*| = 0.285; Figure 9(f)), upon analysis.

## 4. Discussion

As mentioned in our previous meta-analysis [27], cancer still poses a serious threat to human health, and the incidence of cancer is gradually increased in recent years [1]. However, the exact mechanism of metastasis remains unclear in cancer patients despite the fact that the occurrence of metastasis is an important indicator of poor prognosis for patients [28, 29]. On this account, it is necessary to identify the new molecular markers to predict tumor metastasis, which have been found to play critical roles in cancer treatment and prediction [30]. lncRNAs are one of these molecular markers, which can affect tumor genesis and development, and can easily collect biomarkers useful for monitoring and diagnosing tumors [31].

According to previous studies, lncRNA GHET1 has been proved to be an important oncogene in various human cancers, including NSCLC, HCC, BC, ESCC, HNC, BRC, GC, and PC [17–26]. It can be found based on the literature that lncRNA GHET1 may play an important role in cancer progression, which is achieved through regulating the LATS1/YAP signaling pathway and epithelial-mesenchymal transition (EMT). Additionally, it is confirmed based on recent studies that lncRNA GHET1 expression is aberrantly high in HCC tissues and that lncRNA GHET1 silencing can inhibit the migration, proliferation, invasion, and EMT of HCC cells [32]. Moreover, the study by Shen et al. suggested that lncRNA GHET1 expression was remarkably higher in lung NSCLC specimens than in paracarcinoma tissues, and the progression-free survival (PFS) and OS of lncRNA NSCLC patients with lncRNA GHET1 overexpression are shorter than those of the patients with low expression [22]. lncRNA GHET1 has been proved that it could promote osteosarcoma and cervical cancer development and progression via Wnt/*β*-catenin signaling pathway [33, 34], and WNT might be an important marker for targeted cancer therapy [35].

Drug resistance is the main obstacle to successful chemotherapy for patients with cancer. The relationship between lncRNA GHET1 and drug resistance has been studied in gastric cancer and other solid tumors. It is to provide preclinical data for the translational research of lncRNA GHET1 in cancer treatment. Li et al. [36] found that high expression of lncRNA GHET1 was related with the low sensitivity to gemcitabine of BC, lncRNA GHET1 contributed to chemotherapeutic resistance to gemcitabine in BC through upregulating ABCC1 expression. Wei et al. summarized that ncRNAs may be a new therapeutic target and prognostic biomarker for GC [37]. And Zhang et al. reported that high lncRNA GHET1 expression could promote the development of multidrug resistance which was related to the Bax, Bcl-2, MDR1, and MRP1 gene expression in GC cells [38]. Besides, previous studies have shown that there is a specific regulatory relationship between lncRNA GHET1 and c-Myc in GC [11, 39]. Myc is a nuclear transcription factor that mainly regulates cell growth, cell cycle, metabolism, and survival. A number of studies have reported that c-Myc is closely related to the drug sensitivity of GC [40, 41]. These results suggest that lncRNA GHET1 and its associated molecules are closely related to tumor progression and drug sensitivity. Since the role of the hypoxic tumor microenvironment in chemotherapy failure has been increasingly considered in GC and other tumors recently [42,43], this might be a further direction to study the mechanism of lncRNA GHET1 regulating tumor drug resistance.

To further investigate the underlying mechanism, target, and function of lncRNA GHET1 in cancer, the potential targets, pathways, and related miRNA of lncRNA GHET1 had been systematically reviewed (Table 4). As mentioned above, the expression of lncRNA GHET1 is closely related to OS and clinicopathological characteristics. The reason may be that lncRNA GHET1 is closely related to P21, c-Myc, WNT, and other tumor proliferation, invasion, and prognosis molecular markers. However, the underlying mechanisms by which lncRNA GHET1 influenced the cancer remained unknown yet. This meta-analysis had discussed the prognostic value and clinicopathological significance of lncRNA GHET1 in cancer patients.

In this meta-analysis, data collected from the eleven eligible studies involving seven hundred and fourteen cancer patients were analyzed. Subsequently, a fixed- or a random-effects model was adopted depending on the results of heterogeneity analysis. For cancer patients, high lncRNA GHET1 expression might potentially serve as an indicator of poor prognosis. A significant difference in OS was detected between the high and low lncRNA GHET1 expression groups after pooling HRs from Cox multivariate analyses. In addition, high lncRNA GHET1 expression was found to be dramatically correlated with poor OS in various cancer types. Furthermore, high lncRNA GHET1 expression was also remarkably related to the clinicopathological parameters in cancer patients, including LTS, PHG, HTS, LNM, and DM. To sum up, findings in this study indicated that lncRNA GHET1 might serve as a valuable prognostic biomarker for the poor prognosis of most cancers.

## 5. Strengths and Limitations

When interpreting the conclusions of the current meta-analysis, several limitations should be taken into consideration. Firstly, all studies included in this meta-analysis were retrieved from three online databases; as a result, it was likely that some related papers might be missed. Secondly, after searching the database according to the inclusion and exclusion criteria, only ten studies came from China, so more research from other countries in the future is still needed to prove our conclusions. Thirdly, the cutoff values of the high- and low-expressed lncRNA GHET1 in these studies were not consistent. In addition, as bone spreading is also an important clinical feature related to tumor progression and prognosis, the role of lncRNA GHET1 in tumor-associated bone skeletal diffusion can be studied in the future. Finally, other factors that may affect cancer prognosis, such as treatment and tumor subgroup, were not included in this study. Because these included studies do not include these data, this may be an inherent shortcoming of this systematic review and meta-analysis.

## 6. Conclusions

To sum up, high lncRNA GHET1 expression in a series of cancers is associated with poor OS, LTS, PHG, HTS, LNM, and DM. As a result, lncRNA GHET1 can be used as a promising biomarker to predict tumor metastasis and prognosis in cancer patients.

## Figures and Tables

**Table 1:** The basic information and data of all included studies in the meta-analysis.

Study	Year	Country	Tumor type	Sample size	lncRNA GHET1 expression												Reference gene	Detection method
					High expression						Low expression							
					Total	LTS	PHG	HTS	LNM	DM	Total	LTS	PHG	HTS	LNM	DM		
Guan et al. [17]	2018	China	NSCLC	52	25	—	16	13	19	—	27	—	15	6	12	—	GAPDH	PCR
Jin et al. [18]	2017	China	HCC	68	27	21	20	—	—	—	41	14	14	—	—	—	GAPDH	PCR
Li et al. [19]	2014	China	BC	80	39	—	—	—	—	—	41	—	—	—	—	—	GAPDH	PCR
Liu et al. [20]	2017	China	ESCC	55	28	16	14	22	12	—	27	10	6	9	4	—	GAPDH	PCR
Liu and Wu [21]	2018	China	HNC	86	43	27	23	—	25	27	43	19	15	—	10	15	GAPDH	PCR
Shen et al. [22]	2018	China	NSCLC	105	53	33	—	28	32	—	52	12	—	13	7	—	GAPDH	PCR
Song et al. [23]	2018	China	BRC	60	30	19	—	21	18	—	30	10	—	11	9	—	GAPDH	PCR
Xia et al. [24]	2018	China	GC	42	21	7	17	16	16	4	21	10	19	9	19	2	GAPDH	PCR
Yang et al. [11]	2014	China	GC	42	21	17	15	—	10	2	21	9	11	—	6	0	GAPDH	PCR
Yang et al. [25]	2018	China	OSC	60	30	15	—	20	19	21	30	12	—	11	9	10	GAPDH	PCR
Zhou et al. [26]	2017	China	PC	64	36	23	26	24	—	6	28	10	14	9	—	0	GAPDH	PCR

*Note.* The dashes represent no data. LTS, lager tumor size; PHG, poor histological grade; HTS, high tumor stage; LNM, lymph node metastasis; DM, distant metastasis; NSCLC, non-small-cell lung cancer; HCC, hepatocellular carcinoma; BC, bladder cancer; ESCC, esophageal squamous cell carcinoma; HNC, head and neck cancer; BRC, breast cancer; GC, gastric cancer; OSC, osteosarcoma; PC, pancreatic cancer; PCR, polymerase chain reaction; GAPDH, glyceraldehyde-3-phosphate dehydrogenase.

**Table 2:** Survival data of studies included in the meta-analysis.

Study	Year	Country	Tumor type	Sample size	Method	Overall survival (OS)	Cutoff value	NOS
Guan et al. [17]	2018	China	NSCLC	52	Multivariate	2.488(1.415–3.841)	Median	7
Jin et al. [18]	2017	China	HCC	68	Multivariate	7.64(2.28–25.65)	Mean	8
Li et al. [19]	2014	China	BC	80	Multivariate	2.59(1.27–5.25)	Median	7
Liu et al. [20]	2017	China	ESCC	55	NA	NA	Median	7
Liu and Wu [21]	2018	China	HNC	86	Multivariate	2.21(1.25–3.89)	Median	8
Shen et al. [22]	2018	China	NSCLC	105	Multivariate	2.20(1.38–3.51)	Median	7
Song et al. [23]	2018	China	BRC	60	Multivariate	1.92(1.06–3.49)	Median	8
Xia et al. [24]	2018	China	GC	42	NA	NA	Median	7
Yang et al. [11]	2014	China	GC	42	Multivariate	1.88(1.03–3.42)	Median	8
Yang et al. [25]	2018	China	OSC	60	Multivariate	2.14(1.13–4.05)	—	7
Zhou et al. [26]	2017	China	PC	64	NA	NA	Median	7

*Note.* NA represent no data. NSCLC, non-small-cell lung cancer; HCC, hepatocellular carcinoma; BC, bladder cancer; ESCC, esophageal squamous cell carcinoma; HNC, head and neck cancer; BRC, breast cancer; GC, gastric cancer; OSC, osteosarcoma; PC, pancreatic cancer; OS, overall survival; NOS, Newcastle–Ottawa scale.

**Table 3:** Subgroup analysis of OS by tumor type, sample size, and NOS score.

Subgroup analysis	No. of studies	No. of patients	Pooled HR (95% CI)	Heterogeneity	
				*I * ^2^ (%)	*P* value
Total	8	553	2.28(1.85–2.82)	0.0	0.676
Tumor type					
Nondigestive system cancer	6	443	2.24(1.78–2.82)	0.0	0.987
Digestive system cancer	2	110	2.48(1.45–4.24)	75.8	0.042
Sample size					
Number <80	5	282	2.28(1.72–3.02)	15.0	0.319
Number ≥80	3	271	2.28(1.65–3.14)	0.0	0.924
NOS score					
NOS>7	4	256	2.21(1.59–3.06)	33.8	0.210
NOS ≤7	4	297	2.33(1.77–3.07)	0.0	0.964

OS, overall survival; NOS, Newcastle–Ottawa scale; HR, hazard ratio; CI, confidence interval.

**Table 4:** Summary of lncRNA GHET1 with their potential targets, related genes, pathways, and function entered this study.

First author	Cancer type	Potential targets	Related genes or pathway	Function
Guan [17]	NSCLC	YAP1	LATS1/YAP signaling pathway	Cell proliferation, invasion, and epithelial-mesenchymal transition (EMT)
Jin [18]	HCC	EZH2	KLF2	Cell proliferation, cycle arrest, and apoptosis
Li [19]	BC	NA	p16, p21,E-cadherin, fibronectin, and vimentin	Cell proliferation, cycle arrest, invasion, and EMT
Liu [20]	ESCC	NA	Vimentin, N-cadherin, and E-cadherin	Proliferation migration, invasion, apoptosis, and EMT
Liu [21]	HNC	NA	NA	Cell proliferation, apoptosis, cycle arrest, migration, and invasion
Song [23]	BRC	NA	Vimentin, N-cadherin, and E-cadherin	Cell proliferation, invasion, migration, apoptosis, cycle arrest, and EMT
Xia [24]	GC	NA	PCNA, cyclin D, CDK4, CDK6, cyclin E, CDK2, and P21	Cell cycle arrest, proliferation, migration, and invasion
Yang [11]	GC	IGF2BP1	c-Myc	Cell proliferation
Yang [25]	OSC	NA	ZEB2, Snail, vimentin, N-cadherin, and E-cadherin	Cell proliferation, invasion, migration, apoptosis, cycle arrest, and EMT
Zhou [26]	PC	NA	NA	Cell proliferation, apoptosis, and cycle arrest

## Data Availability

Meta-analysis is a secondary analysis, in which the data are all fully available without restriction, and all the material can be found in the included original studies.

## Abbreviations

lncRNA GHET1: Long noncoding RNA gastric carcinoma highly expressed transcript 1
LTS: Larger tumor size
PHG: Poor histological grade
HTS: High tumor stage
LNM: Lymph node metastasis
DM: Distant metastasis
NSCLC: Non-small-cell lung cancer
HCC: Hepatocellular carcinoma
BC: Bladder cancer
ESCC: Esophageal squamous cell carcinoma
HNC: Head and neck cancer
BRC: Breast cancer
GC: Gastric cancer
PC: Pancreatic cancer
OS: Overall survival
HR: Hazard ratio
CI: Confidence interval
NOS: Newcastle–Ottawa scale
PCR: Polymerase chain reaction
GAPDH: Glyceraldehyde-3-phosphate dehydrogenase.
